# Cases of Floating Kidneys

**Published:** 1874-01-01

**Authors:** Abram Sager

**Affiliations:** Professor of Obstetrics in University of Michigan


### CASES OF FLOATING KIDNEYS.

REPORTED TO WASHTENAW CO. MEDICAL SOCIETY, SEPT. 23, 1873, BY ABRAM SAGER, M.D., PROFESSOR OF OBSTETRICS IN UNIVERSITY OF MICHIGAN.

*From the Peninsular Journal of Medicine.*

THERE are various anomalies of form, of size, position and connection of the kidneys, which the investigations of the pathological anatomist have revealed to us. These anomalies may affect either one or both of these normally symmetrical organs. Thus they may occupy a position either higher or lower than usual, one of them may be in the pelvic cavity, or on its superior border. They may be more or less completely united and occupy a position upon the side of, or upon the nesial line, over the vertebræ. They may be entirely absent; unequally developed, or a single one of abnormal size; lastly, they may be lobulated, thus exhibiting a fœtal or inferior type of structure. These abnormalities are nearly always of a congenital character, and have their origin in some error of the development process. They are of importance chiefly in a diagnostic point of view, as they may be confounded with tumors of the ovaries, or the uterus, and have, though rarely, impeded the process of parturition. Setting aside these fixed anomalies of the organs, I beg to draw your attention to one variety of position less rarely met with, viz.: movable or floating kidneys. This anomaly affects far more frequently one than both simultaneously. It occurs much more frequently also in women than in men. Various, but not very satisfactory, causes have been assigned for these facts; doubtless in some cases it is of congenital origin, but its more frequent occurrence in woman may be due to the conditions of pregnancy, and tight lacing.

The following brief notes of two cases that have fallen under my observation during the last year, may convey some idea of the symptoms and signs exhibited by them, and also show why they are liable to be confounded with other tumors and growths of the abdomen:

CASE I.—Mrs. M., about thirty-five years old, of rather slender form, and the mother of one child, called on me, and stated that she had some trouble with her side, which gave rise to some pain and uneasiness at times, and often coupled with a dragging sensation when in the erect position.

She had already had the opinion of one or two physicians. They had considerably alarmed her by speaking the name of tumor; a name which, when applied to adventitious or normal growths of the abdomen, is generally associated in the minds of the laity with the anceps remedium. On placing the patient in the dorsal decubitus, and inclining toward the left side, I readily discovered a tumor a little below the margin of the false ribs. It had about the size and the uniform figure of a kidney, and was smooth to the touch. It could be readily moved in various directions, but chiefly upward and backward, and somewhat transversely. It could be depressed to the umbilicus, and slightly across the median line; backward and upward it could be carried completely under the margin of the false ribs; neither manipulation nor pressure caused any considerable pain, but rather a slight nausea or faintness. A slight degree of flatness of the dorsum over the site of the kidney, as compared with normal size; and greater resonance, or rather less dullness or percussion, indicated the absence of the kidney from its usual location. Anteriorly, the presence of the organ but slightly affected the usual resonance over the bowels.

I have seen the patient twice since at intervals of several months, and found no change in the physical signs. Her physician insisted that it was not a kidney, but a floating tumor; but of what nature he did not suggest; and, to confirm her doubts, one of her lady friends assured her it could not be a movable kidney, as she had never heard of such a thing.

I advised her to use an elastic bandage to give some support to the parietes and lessen the sense of dragging on the vessels and pedicle of the kidney; also, what seemed appropriate treatment, partly moral, for the atonic dyspepsia. She reported that the bandage was more uncomfortable than the tension, and hence had discontinued it. Beside a moral tonic regimen, I advised nothing further.

CASE II.—Mrs. S., twenty-six years old, married, and had one abortion, called to consult me in reference to some uterine disease. She was pale and sallow, and suffered from severe gastric dyspepsia. In giving the history of her case she stated she had been treated for enlargement of the liver. On examining the right hypochondrum, I discovered a tumor projecting below the ribs, round, smooth, and firm, and about the size of the kidney. It was not movable to the same extent as in the former case, yet distinctly so. By manipulation the entire tumor could be distinctly felt below the ribs, and so separate as to admit of being partially grasped. It was moderately sensitive, firm, and slightly elastic, and could be completely depressed behind the false ribs. There was more pain and uneasiness than in the former case. The margins of the liver could not be felt by the most careful manipulation. She said that in former years she had been accustomed to lace lightly, but at present could not bear pressure, but chiefly on account of the sensibility of the stomach.

The diagnosis of these tumors is important, as they are liable to be confounded with enlargements of the liver and spleen; with mesenteric fibroma; with enlargement of the bile cyst; with hydatid of the liver; mesentery or omentum; and, when they descend very low, with ovarian tumors, especially when, as occasionally happens, the floating kidney is affected with hydronephrosis.

The differential diagnosis must be drawn from the size, especially when observed at various periods; the form; the smoothness and insensibility, except when firmly pressed; and especially with the condition of the free mobility in the direction of the primary location of the organ. Something may also be inferred from the history and progress of the case, especially when considered in reference to cystic enlargement.

But, as in case second, the shape is not always uniform. This happens when the hilus of the kidney is turned backward, giving it a rounded form, the dorsum of the organ being turned anteriorly. The mobility, the firmness, and the absence of distinct tenderness, are here the chief criteria of diagnosis. From mesenteric fibroma, except by the fosm, mobility, and constancy of size and position, it could with difficulty be distinguished from ovarian tumor; the direction of its motion, passing from below upward and backward, would easily differentiate it.

There being, for itself, little required in the line of treatment, I repeat that it is chiefly to avoid unnecessary or injurious medication, that the diagnosis becomes important.

